# Basic properties mapping of anodic oxides in the hafnium–niobium–tantalum ternary system

**DOI:** 10.1080/14686996.2018.1498703

**Published:** 2018-08-13

**Authors:** Andrei Ionut Mardare, Cezarina Cela Mardare, Jan Philipp Kollender, Silvia Huber, Achim Walter Hassel

**Affiliations:** a Institute for Chemical Technology of Inorganic Materials, Johannes Kepler University Linz, Linz, Austria; b Competence Centre for Electrochemical Surface Technology, Wiener Neustadt, Austria; c Christian Doppler Laboratory for Combinatorial Oxide Chemistry, Institute for Chemical Technology of Inorganic Materials, Johannes Kepler University Linz, Linz, Austria

**Keywords:** Combinatorial libraries, high-throughput experimentation, scanning droplet cell microscopy, anodic oxide films, valve metals, 40 Optical, magnetic and electronic device materials, 202 Dielectrics / Piezoelectrics / Insulators, 306 Thin film / Coatings, Anodic oxides

## Abstract

A thin film combinatorial library deposited by co-sputtering of Hf, Nb and Ta was employed to characterise fundamental properties of the Hf-Nb-Ta system. Compositional mappings of microstructure and crystallography revealed similarities in alloy evolution. Distinct lattice distortion was observed upon addition of hexagonal Hf, leading to amorphisation of alloys containing more than 32 at.% Hf and less than 27 and 41 at.% Nb and Ta, respectively. Volta potential and open circuit potential mappings indicated minimal values for the highest Hf concentration. Localised anodisation of the library by scanning droplet cell microscopy revealed valve metal behaviour. Oxide formation factors above 2 nm V^−1^ were identified in compositional zones with high amounts of Nb and Ta. Fitting of electrochemical impedance spectroscopy data allowed electrical permittivity and resistivity of mixed oxides to be mapped. Their compositional behaviours were attributed to characteristics of the parent metal alloys and particularities of the pure oxides. Mott–Schottky analysis suggested n-type semiconductor properties for all Hf–Nb–Ta oxides studied. Donor density and flat-band potential were mapped compositionally, and their variations were found to be related mainly to the Nb amount. Synergetic effects were identified in mappings of Hf-Nb-Ta parent metals and their anodic oxides.

## Graphical Abstract

**Figure:**
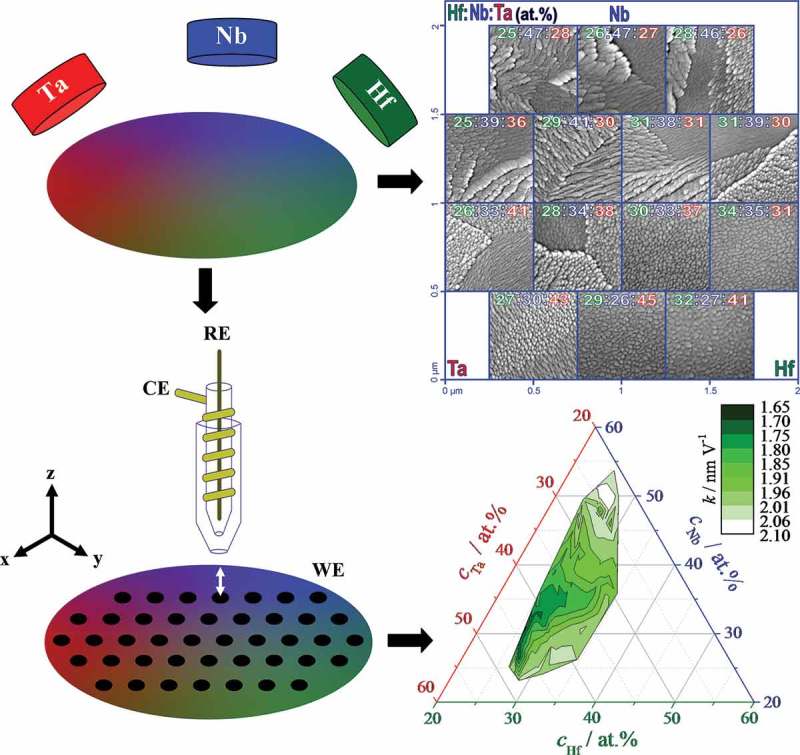

## 1. Introduction

Combinatorial analysis in materials research is becoming essential not only to the development of new materials with improved properties, but also in identifying alloys whose special properties have been overlooked as a result of insufficient compositional resolution, that is, an insufficient number of samples prepared and/or analysed. Such approaches rely on high-throughput systems that are capable of reproducibly addressing one alloy at a time, usually in an automatic scanning procedure [1,2]. The use of thin film combinatorial libraries is a convenient solution for obtaining a high number of alloys with identical history on the same substrate [3–6]. Following the compositional evolution of a given property (i.e. screening for an interesting feature) provides insights into the causes for property changes and allows possible applications to be identified [7–10].

Hafnium, niobium and tantalum have similar electrochemical characteristics in that they are all valve metals. This classification and its name are based on their current rectification upon electric field reversal (hence, the name ‘valve’) during metals anodisation under high field conditions. The final anodic oxide thickness is proportional to the applied potential and oxide formation factors of 2.3, 2.6 and 1.8 nm V^−1^ were measured for Hf, Nb and Ta, respectively [11]. The oxides of the aforementioned pure metals have applications in various fields. Due to its high dielectric constant and excellent thermal stability, hafnium oxide is investigated mainly as a gate material for field effect transistors and supercapacitors, while hafnium oxynitride is studied as a catalyst for oxygen reduction reactions [12,13]. Additionally, Hf_1__−_ *_x_*Ta*_x_*O_2_ based memristors with excellent bipolar resistive switching characteristics were recently demonstrated, which promote the use of mixed Hf and Ta oxides in modern electronics [14]. The spectrum of niobium oxide applications is broad, ranging from use in capacitors to applications in electrochromic devices, gas sensors and solar cells [15–18]. Applications of Ta_2_O_5_ are found in the same major areas. Tantalum oxides, usually applied in high power resistors and capacitors, started recently to be investigated for cathodes in fuel cells, lithiation support in batteries and counter electrodes in solar cells [19–22].

Combining these oxides may result in materials with useful properties for new applications. Anodisation of binary thin film combinatorial libraries containing Hf, Nb or Ta has already revealed very interesting features. Screening of a Hf–Nb library showed the effect of Nb as a cubic phase stabiliser in metallic alloys and a remarkably high electrical permittivity of mixed Hf–Nb anodic oxides [23]. Semiconducting properties of Nb_2_O_5_ were imprinted on mixed oxides grown anodically on Nb–Ta combinatorial libraries, with relatively high charge carrier densities, while the dielectric constants remained at high values [24]. Screening of a Hf–Ta combinatorial library showed strong amorphisation of the parent metal alloys, which had a direct impact on the electrochemical properties of the mixed anodic oxides [25].

This work focuses on property mapping in the Hf–Nb–Ta ternary system. In addition to characterising the parent metallic alloys compositionally, the anodic oxide formation is studied and the properties of mixed Hf–Nb–Ta oxides are mapped for the development of future applications.

## 2. Experimental section

### 2.1. Thin film deposition

A thin film combinatorial library containing Hf, Nb and Ta was deposited using a confocal co-sputtering system (Mantis Deposition GmbH, Thame, UK) with 50 mm (in diameter) targets. A thermally oxidised Si wafer (100 mm in diameter, Si-Mat, Kaufering, Germany), with approximately 2 µm thick SiO_2_, was used as substrate. Three different sputtering targets (Hf – Demaco, Noord-Scharwoude, The Netherlands, Nb – MaTeck Material-Technologie & Kristalle GmbH, Jülich, Germany and Ta – HMW Hauner GmbH & Co. KG, Röttenbach, Germany) with 99.95% purity, positioned at 120° relative to each other were used simultaneously for depositing the Hf–Nb–Ta compositional spread. The deposition angle was 54°, the target–substrate distance was 130 mm and the deposition rates for Hf, Nb and Ta were 4.8 × 10^−3^ nm W^−1^ s^−1^, 2.4 × 10^−3^ nm W^−1^ s^−1^ and 3.6 × 10^−3^ nm W^−1^ s^−1^, respectively. The sputtering system is designed to uniformly cover substrates up to 100 mm in diameter by using each target. When rotation of the substrate is involved, thickness uniformity (across the substrate) with deviations below 5% is easily achievable. However, since the present study aims at fabricating a combinatorial library, no rotation of the substrate was used. In this way, the vapour phase mixing of Hf, Nb and Ta species allowed forming a ternary compositional gradient. A total thickness of 300 nm was estimated at the centre of the library based on the individual deposition rates, which had previously been calibrated using single metal depositions followed by thin film thickness measurements via contact profilometry (Dektak Billerica, MA, USA). Additionally, 300 nm thick pure Hf, Nb and Ta films were deposited separately under identical conditions (on identical substrates) for reference. The base pressure of the co-sputtering chamber was in the lower range of 10^−6^ Pa and the Hf–Nb–Ta library was deposited in 5 × 10^−1^ Pa Ar atmosphere at room temperature.

### 2.2. Parent metal alloys characterisation

Immediately after the Hf–Nb–Ta thin film deposition, the substrate with the library was transported without breaking vacuum by a robotic arm to a self-developed scanning energy-dispersive X-ray (EDX) system for compositional mapping. Using a non standard electron column without magnetic scan option, the EDX mapping was performed with high spatial resolution across the entire surface. Automatic surface scanning using a vacuum-compatible gantry robot was controlled by LabView (National Instruments Corporation, Austin, TX, USA) software. The primary electron beam was accelerated to 20 keV and the spot size was set at 500 µm in order to enhance the signal-to-noise ratio for thin film analysis. A Si drift detector (SDD, remX GmbH, Bruchsal, Germany) was employed for X-ray detection, and IDFix software (from remX GmbH) was used for data acquisition and quantification. Definition of scanning areas and the automatic control of the IDFix software were preformed using a self-developed LabView program. A compositional map of the library was created that related Hf–Nb–Ta composition to position/surface location.

The Hf–Nb–Ta library was mapped microstructurally and crystallographically before anodic oxide formation using scanning electron microscopy (SEM) and scanning X-ray diffraction (XRD), respectively. To this end, a field emission Zeiss (Oberkochen, Germany) Gemini 1540 XB SEM (with 20 kV acceleration voltage and in-lens detection) and a Philips (Almela, The Netherlands) X’pert Pro XRD system operated in grazing incidence mode (0.4° angle, Cu-Kα source) were used. Volta potential mapping of the parent metal alloys was performed by an in-house developed scanning Kelvin probe (SKP) system (employing a SKP measuring head from Wicinski-Wicinski GbR, Erkrath, Germany) built into a customised climate stabilisation chamber. The SKP probe material was a NiCr alloy with a tip diameter of 300 µm. The vibration frequency was approximately 1 kHz and the mean tip–sample distance was set to 75 µm. The relative humidity inside the climatic chamber was 11 ± 1%, and the ambient temperature was 22 ± 1 °C. Before mapping, the potential of the system was calibrated with saturated aqueous solution of CuSO_4_ in a Cu crucible. All potentials are relative to the standard hydrogen electrode (SHE).

Electrical resistivity measurements were performed across the Hf–Nb–Ta compositional spread using a 4-point probe method. The library was screened using an XYZ stage/scanner developed in-house and controlled by LabView. A cylindrical measuring head (Jandel, Linslade, UK) holding 4 WC needles with a tip diameter of 25 µm and in-line spacing of 635 µm was used for this purpose. A sourcemeter (Keithley 2410) and a multimeter (Keithley 2750 Solon, OH, USA) were used for sourcing the current (through the two outermost needles) and measuring the potential drop (at the two innermost needles), respectively. The value of applied current was varied from 0 to 100 mA in 10 mA steps. The value of the resistivity was calculated from the linear *I*–*V* dependence, before the Joule heating would induce observable resistance changes. The needles were pressed concomitantly onto the library surface with a force of 5 N. A total number of 291 locations uniformly distributed across the library surface and resembling the circular sample geometry (similar to the SKP measurements) were analysed by the LabView software.

### 2.3. Scanning droplet cell microscopy and anodic oxides formation

**Figure 1.**
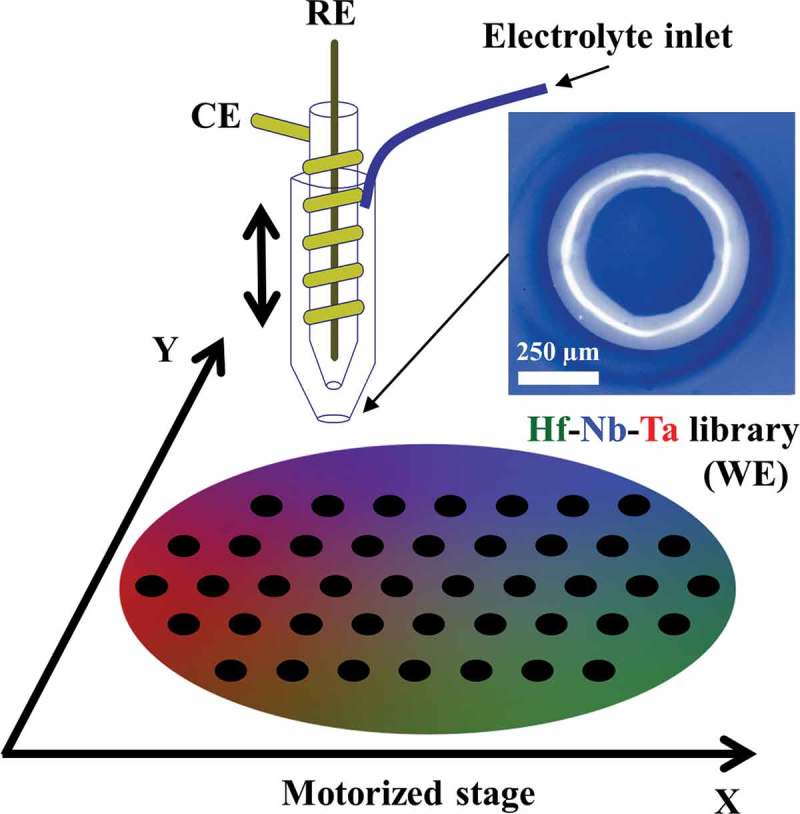
Schematic of library screening using SDCM. RE stands for reference electrode, CE for counter electrode and WE for working electrode. Right inset shows an optical image of the tip with silicone sealing.

The scanning droplet cell microscopy (SDCM) technique was used for all electrochemical investigations presented in this work [26,27]. Based on the concept of bringing a small electrolyte droplet in contact with surface under investigation, SDCM was used in contact mode that allowed surface localisation [28]. Mapping of basic properties along the Hf–Nb–Ta thin film combinatorial library was performed by attaching the SDCM to a home-built gantry robot controlled by LabView software [29,30]. Schematic of the experimental approach is shown in Figure 1, where black circles indicate measurement points.

The microelectrochemical cell (serving as the SDCM measuring head) was completed by joining several parts fabricated separately. First, the cell body was obtained by thermally pulling a borosilicate glass capillary, 2.5 mm in diameter (PC-10, Narishige, Tokyo Japan). The tapered tip produced was ground to a final diameter of 500 µm and then terminated by a silicone soft-sealing formed in a dip/dry process. An optical image of the tip with the silicone sealing is presented in Figure 1. Second, a µ-reference electrode (µ-RE, AuHg/Hg_2_(CH_3_COO)_2_/NaCH_3_COO) was fabricated by electrodeposition of Hg_2_(CH_3_COO)_2_ on an amalgamated Au wire with a diameter of 100 µm [31]. The wire was inserted into a new capillary (1 mm in diameter) that was filled with NaCH_3_COO solution containing 2 wt% agar for conferring mechanical stability. Third, a counter electrode was fabricated by wrapping an Au wire (100 µm) around the µ-RE glass capillary. These three components (schematically represented in Figure 1) were mounted together in an acryl block (not shown here) endowed with an electrolyte inlet for the control of droplet release. Silicone tubes were used in combination with a peristaltic pump (constructed in house) for electrolyte control.

Localised anodic oxides were grown potentiodynamically by SDCM along the Hf–Nb–Ta thin film combinatorial library with a potential increase rate of 100 mV s^−1^ using a CompactStat Potentiostat (Ivium Technologies, Eindhoven, The Netherlands). The thickness of the anodic oxides was gradually increased from 0 to 10 V (SHE) in 1 V steps, which enabled thickness (and composition) dependent oxide impedance measurements. The integrated frequency response analyser was used for this purpose in a frequency range of 10^6^ to10^−1^ Hz. The impedance data was fitted in batch mode using the ZView software from Scribner Associates Inc. (Southern Pines, NC, USA). For assessing the semiconducting properties of the mixed Hf–Nb–Ta anodic oxides, Mott–Schottky analysis was performed on oxide spots previously anodised at 3 V (SHE) by varying the applied DC bias between −1 and 3 V (SHE). For all electrochemical measurements, analytical grade acetate buffer solution (CH_3_COOH/CH_3_COONa) with a fixed pH value of 6.0 was used as electrolyte.

## 3. Results and discussion

**Figure 2.**
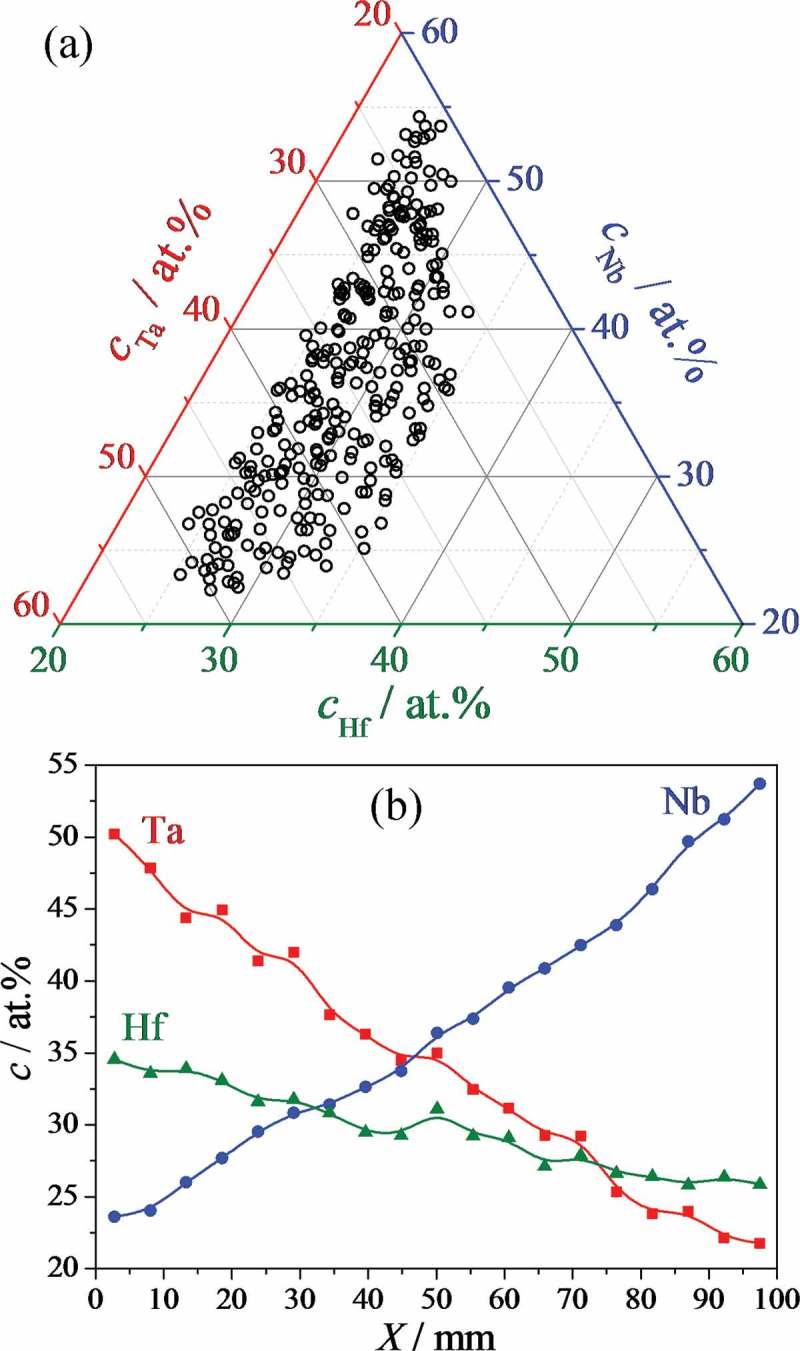
Compositional spread of Hf–Nb–Ta thin film combinatorial library represented as ternary diagram (a) and individual component gradients (b).

Prior to electrochemical experimentation, compositionally induced microstructural and crystallographic particularities of various Hf–Nb–Ta alloys were mapped across the library. The compositional spread itself was first mapped by scanning EDX immediately after deposition (without breaking vacuum), the results of which are presented in Figure 2. For better understanding, all graphical representations in this article use the same colour code for Hf (green), Nb (blue) and Ta (red). In the ternary diagram presented in Figure 2(a), it can easily be seen that across the 100 mm Si wafer a broad compositional mixture of Hf, Nb and Ta was obtained. The experimental points indicate alloys, uniformly distributed across the entire surface, containing between 25 and 35 at.% Hf, between 23 and 54 at.% Nb and between 21 and 51 at.% Ta.

As this ternary diagram provides no information about the location of any particular alloy on the Hf–Nb–Ta library, Figure 2(b) shows the individual Hf, Nb and Ta gradients as measured along their highest slopes, which coincide with the deposition directions of the sources (i.e. with 120° angles in between). The individual gradients for each element are almost linear, which is due to the chosen (confocal) sputtering geometry combined with a weak cosine law responsible for elemental thickness uniformity distribution. Each individual compositional gradient allows the elemental compositional resolution obtained to be measured: 0.1 at.% mm^−1^ for Hf and 0.3 at.% mm^−1^ for Nb and Ta. This indicates that identifying a specific Hf–Nb–Ta thin film alloy at a typical precision of 1 at.% translates to an area 10 mm along the Hf gradient and approximately 3 mm along the Nb and Ta gradients on the library surface – a conveniently large area to be probed simultaneously in further mappings described in this study. This information is essential because interference from neighbouring alloys is to be kept to a minimum at a single location on the surface.

**Figure 3.**
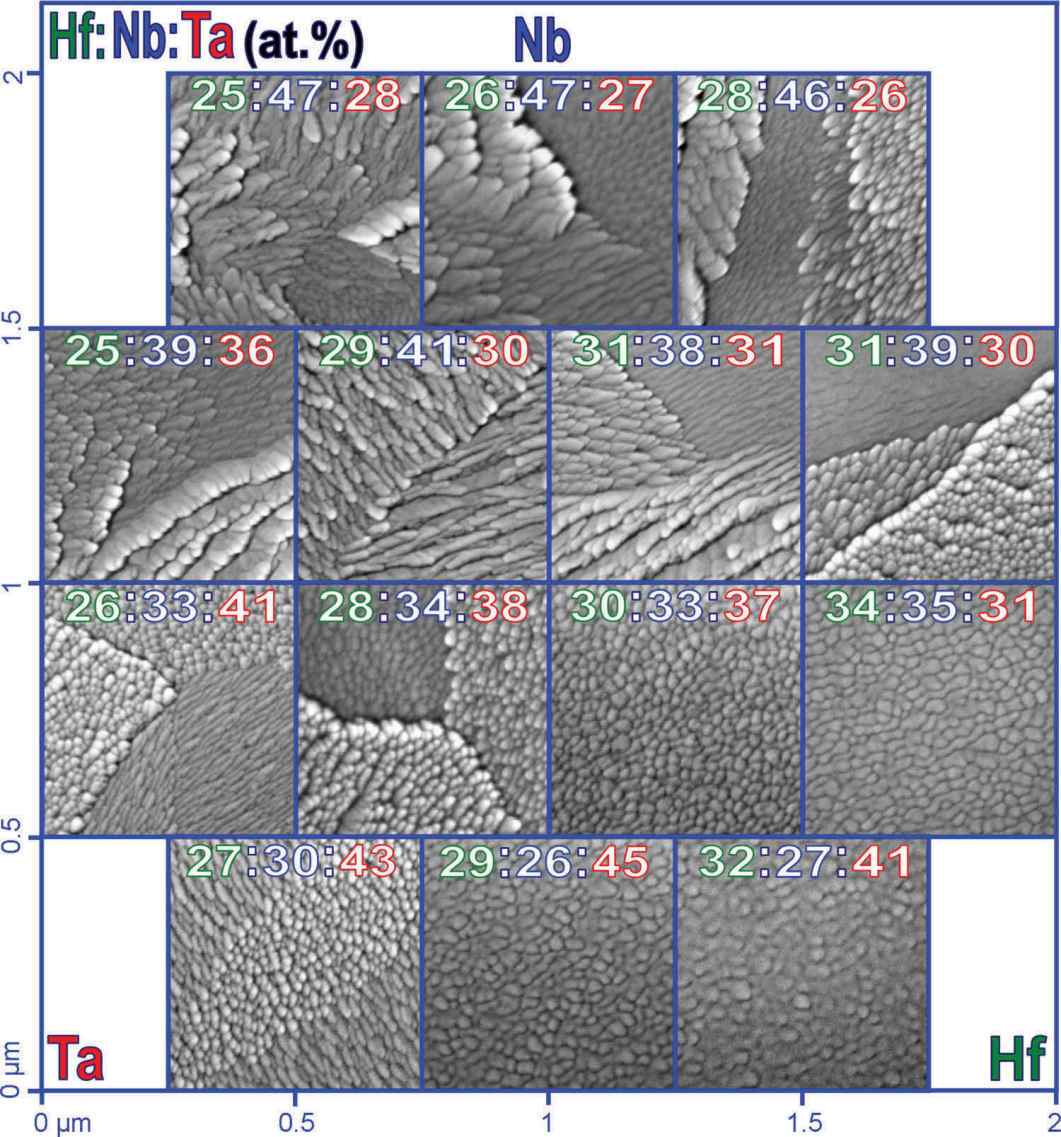
Selected SEM images describing relevant microstructure changes across the Hf–Nb–Ta thin film combinatorial library.

Microstructural mapping of the Hf–Nb–Ta ternary library was performed by SEM. In this process, the surfaces of various alloys were imaged according to their compositions (using EDX mapping). Figure 3 presents a table of selected SEM images of Hf–Nb–Ta thin film alloys uniformly distributed across the circular surface of the library. The position of each elemental deposition source is indicated in the table and all atomic ratios are presented in each image as colour-coded. The alloys in the top SEM row, where Hf, Nb and Ta have approximately 1:2:1 ratios, exhibit similar features. Slightly elongated surface grains, with about 100 nm equivalent diameter, are grouped in domains for high Nb amounts and almost equal Hf and Ta concentrations. This type of microstructure has previously been observed in both Nb–Ta and Hf–Nb binary thin film libraries (for Nb amounts around 66 at.%) which indicate that surface domains formation may be triggered by the presence of Nb [23,24]. Additionally, the study of a Hf–Ta binary library revealed that amorphisation of alloys occurs at equal amounts of Hf and Ta [25].

The presence of surface domains dominates most of the microstructure of the Hf–Nb–Ta thin film library, as can be seen in Figure 3. The overall grain size decreases with decreasing Nb amount (vertically, top/down in Figure 3) and grain shape changes from elongated to round, as shown in the bottom SEM row. Following the increase of Hf amount (diagonally, top-left/bottom-right), a complete disappearance of domains is observed above approximately 30 at.% Hf. However, it may be difficult to isolate such effect to a single element gradient since the entire microstructure depends on all three species present which try to accommodate in a common (minimum energy) atomic arrangement. Nevertheless, almost equal amounts of Hf, Nb and Ta present in the library (bottom-right corner of the SEM table) clearly resulted in a finer surface grain structure that differed from those of other alloys present. This is interpreted as a result of the microstructure screening proposed as part of the current study.

Along the Ta concentration gradient, with the Ta amount increasing from 26 to 43 at.%, grain refinement can also be observed (Figure 3, diagonally, top-right/bottom-left). The surface domains do not disappear, but are strongly suppressed at high Ta concentrations (e.g. 43 at.%) where the domains are defined by alternating regions of small round and longer in-plane grains. Since the Hf–Nb–Ta thin film library was deposited by sputtering, the energy received by each atomic species in the plasma was relatively high (of the order of 100 eV), which resulted in the formation of a typical columnar structure during film growth. The evolution of the surface grain structure across the library thus corresponds to the visible top parts of the columns and their alignments normal to the film surface. Surface grain elongations observed in the second row of the SEM table (Figure 3) describe the vertical inclination of such columns, while changes in surface grain size or shape (e.g. low/high Nb amount) directly describe morphological modifications of the columns. The columns inclination depends on the Hf–Nb–Ta composition across the library since incoming Hf, Nb and Ta species arrived at the same deposition location on the surface from different directions (dictated by the sputtering guns positions). However, from the surface point of view the previous discussion of surface grains evolution (triggered by columns dynamics) is most relevant. This is due to the fact that any follow-up surface modification (e.g. via anodisation) is exclusively surface sensitive.

**Figure 4.**
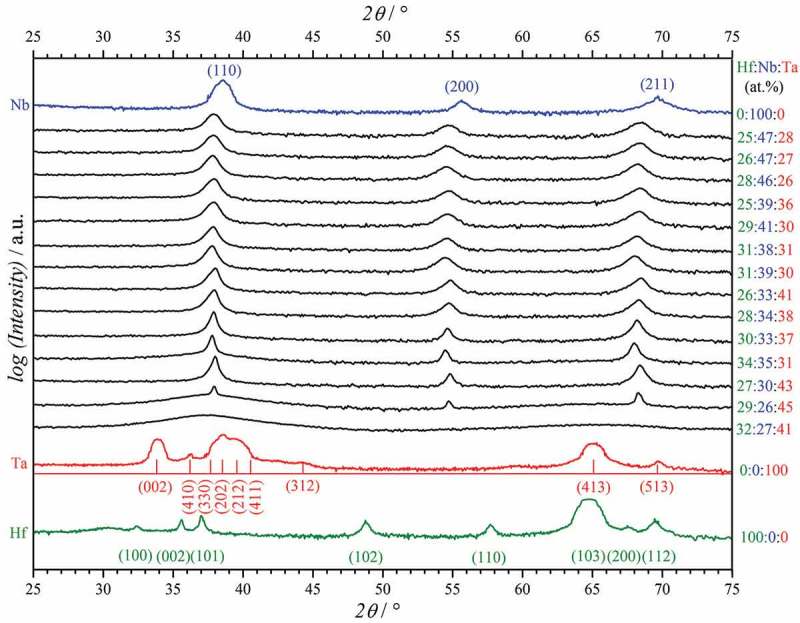
Selected X-ray diffractograms measured across the Hf–Nb–Ta thin film combinatorial library.

The basic properties characterisation of the Hf–Nb–Ta metallic alloys was completed by crystallographic properties mapping. This was performed across the entire compositional spread by scanning XRD and selected diffractograms are presented in Figure 4 as a function of composition. The same Hf–Nb–Ta compositions, which are previously imaged in Figure 3 by SEM, are shown. For referencing purposes, the results obtained (under identical measuring conditions) on pure Hf, Nb and Ta thin films are presented (and colour-coded) in the same figure. The library under study describes a highly interesting crystallographic mixture obtained by combining the hexagonal phase of pure Hf with the cubic phase of pure Nb and the tetragonal phase identified for pure Ta. Such mixture is expected to result in stress build-up within the alloy thin film which in turn will lead to a shift in the XRD peak positions. Different species (Hf, Nb, Ta) coming to the same surface location will show an initial tendency of forming their natural structure with minimum energy defined by their particular characteristics (atomic size, surface mobility on the used substrate, electronegativity, etc.). This tendency is hindered by the presence of alloying species, and in its final state the alloy film may stabilise in a crystalline structure or may suffer different degrees of disordering. Such a peak shift is easily observable, for example when comparing pure Nb (in blue) with most of the ternary alloys (in black) in the diffractograms presented in Figure 3. Observing the individual positions and angular distribution of the three diffraction peaks measured across the Hf–Nb–Ta library reveals a cubic symmetry for most of the ternary alloys. An exception is found in the compositional region where grain refinement and domain disappearance were previously identified in the SEM analysis (lower-right corner of Figure 3). The diffractogram corresponding to Hf:Nb:Ta atomic ratios of 32:27:41 shows a clear case of amorphisation, with a typical very broad peak at low angles.

The amorphisation of Hf–Nb–Ta thin film alloys may explain the surface microstructure smoothing observed in the SEM analysis. Very similar microstructures have previously been observed for binary atomic mixing of Hf and Ta [25]. The transition between hexagonal Hf and tetragonal Ta occurs through a relatively large amorphous compositional zone. However, mixing the cubic phase of Nb with either tetragonal Ta or hexagonal Hf results in very narrow amorphous regions, as binary studies have shown [23,24]. Depending on the composition, the transitions between cubic and either tetragonal or hexagonal phases are sharp. In the ternary library under study, cubic phase stabilisation occurs likely due to the higher symmetry (lower energy) of this phase where each species contributes to the final lattice. The known cubic phase stabilisation effect of Nb is a decisive factor here [32,33]. The observed amorphisation, however, is triggered mainly by mismatches between the three individual symmetries. Such mismatches may have an extremely strong effect on the final symmetries as previously described in the analysis of Hf–Ta–Ti alloys where no clear symmetry group could be found [34]. This suggests that synergetic effects may influence both microstructure and crystallography of such ternary thin film alloys.

**Figure 5.**
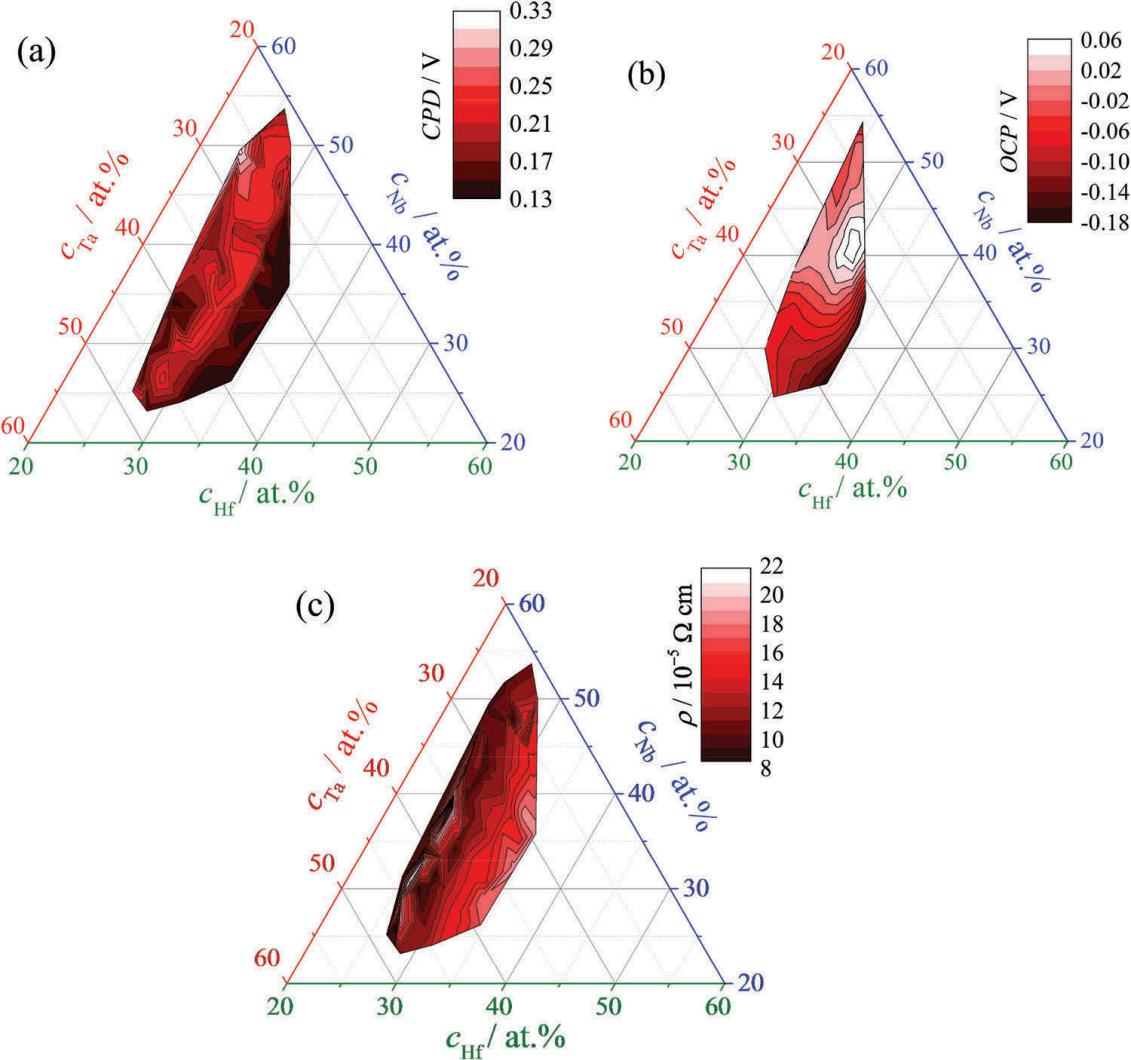
Contact potential difference (a) open circuit potential (b) and electrical resistivity (c) mapping of metallic alloys across the Hf–Nb–Ta combinatorial library.

The scanning Kelvin probe technique was employed in the Volta potential (contact potential difference – CPD) mapping of the ternary library under study. These results are shown in Figure 5(a) as a ternary diagram with colour-coded compositional axis. The measured CPD values show a variation of almost 0.20 V between different alloys in the Hf–Nb–Ta system. This variation is significantly larger than the normal CPD noise of approximately ±5 mV measured above a homogeneous metallic surface [35,36]. The Volta potential of individual alloys across the library depends mainly on the Hf concentration. Values as low as 0.13 V were measured for most alloys containing between 32 and 35 at.% Hf while alloys with approximately 30 at.% Hf showed a CPD increase towards 0.25 V. Low Hf amounts triggered isolated decreases in the CPD influenced by the other species present in the library. Alloys with compositions above 45 at.% Nb but below 30 at.% Ta deviated from the main trend of decreasing Volta potentials induced by an increasing Hf amount. A maximum CPD of 0.33 V was found at a composition of 24 at.% Hf, 49 at.% Nb and 27 at.% Ta, describing a relatively sharp change in this compositional region.

Scanning droplet cell microscopy (SDCM) was used for locally addressing various Hf–Nb–Ta alloys during electrochemical properties mapping of the library. Before anodic oxide growth, the open circuit potential (OCP) of the library surface in contact with the electrolyte was measured. The resulting OCP compositional mapping is presented in Figure 5(b) in a similar manner to the CPD presentation. The OCP compositional evolution is better defined as compared to the previous CPD mapping. A zone centred at approximately 30 at.% Hf, 42 at.% Nb and 28 at.% T can be clearly identified where a maximum OCP of 0.06 V SHE was measured. In both directions along the Ta compositional axis (red) the OCP decreases until a minimum value of −0.18 V (SHE) is reached for the highest Hf amount in the library. Compositional changes along the Hf axis, however, are much smoother, and regions with constant OCP can be found above 30 at.% Nb.

Using a 4-point technique, the electrical conductivity of the Hf–Nb–Ta library was compositionally mapped as well and the results are plotted in Figure 5(c). Due to the sharp needles used for this measurement, it may be assumed that the naturally formed surface oxide is penetrated and the electronic transport is performed through the metallic alloys. The mapping of electrical resistivity shows a similar trend when compared with the CPD and OCP mappings. The main element influencing the charge transport through the metallic alloys is Hf. Overall, an increase in the Hf amount of approximately 10 at.% (from left to right in Figure 5(c)) leads to three times higher resistivity, with Nb and Ta minor influences. However, localised compositional regions may be observed where this trend does not apply as observed at Hf:Nb:Ta ratios of 25:38:37 where the highest electrical conductivity was measured.

The Volta potential of a surface can usually be linked directly to its OCP [37]. However, the surface morphology may play a crucial role in the successful correlation of the two potentials, since the Volta potential is influenced significantly by microstructure [38]. The CPD and OCP mappings presented in Figure 5 indicate that their compositional variations are indeed affected by different factors. In accordance with the SEM analysis (Figure 3), it can be confirmed that the CPD compositional variation is influenced mainly by surface morphology. The microstructure transition towards smoother surfaces due to amorphisation of the Hf–Nb–Ta alloys (above approximately 30 at.% Hf) triggers an almost 50% drop in the CPD, which depends weakly on the amounts of the other species. The increase of Hf amount coincides with formation of smaller, almost round surface grains. A vertical alignment of the structural columns obtained during the film growth would result in exactly this surface appearance. Additionally, the surface electronic transport will be directly affected having as a result a decreased surface nobility of the alloy at high Hf contents. The OCP, however, is affected mainly by the reactivity of the surface in contact with the electrolyte, which is in turn defined primarily by alloy composition. This may be the reason why a defined zone around 30 at.% Hf, 42 at.% Nb and 28 at.% Ta shows the maximum OCP, and its compositional evolution is affected more significantly by variation of all species and not only by that of Hf. One feature is shared by all mappings presented in Figure 5: the CPD, OCP and electrical conductivity values are minimal when the Hf concentration is highest.

**Figure 6.**
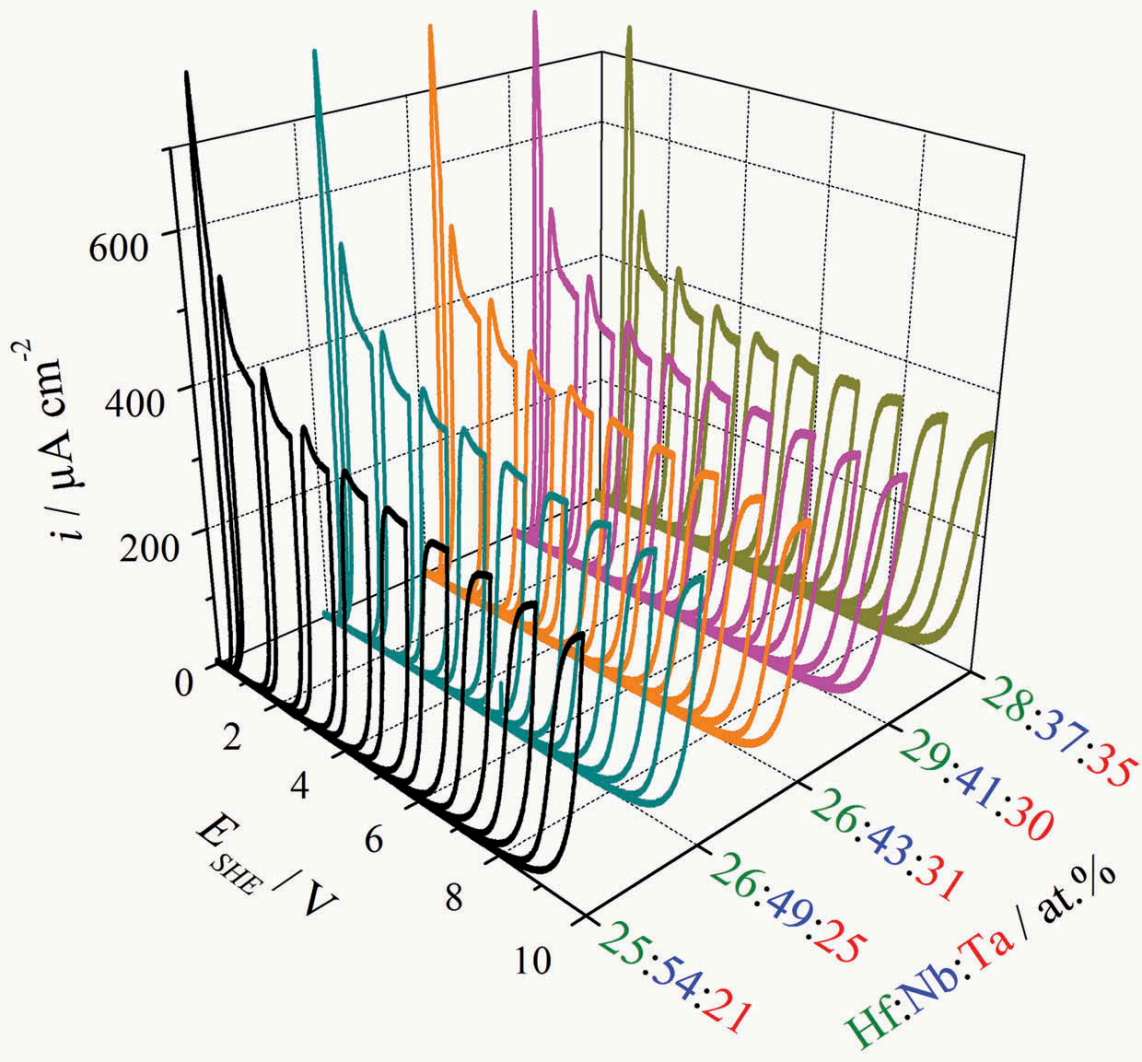
Potentiodynamic oxide formation (100 mV s^−1^) for various Hf–Nb–Ta alloys across the library.

Having detailed mappings of basic properties of the metallic Hf–Nb–Ta alloys, the anodic oxide formation was studied next using scanning droplet cell microscopy (SDCM). The scanner addressed different parent metal alloys, and the anodic oxide was grown step-wise to enable thickness-dependent analysis across the library. Several selected cyclic voltammogram (CV) series are exemplified in Figure 6. Each series contains CVs in which the maximum anodisation potential was step-wise increased between 1 and 10 V SHE. Due to the valve metal nature of all species mixed in the library, a typical current rectifying behaviour upon electric field reversal is clearly observable [11]. Ionic species injection into the initial oxide is best observed during the first anodisation step to 1 V (SHE). Here, current overshoots describing the overlapping of both anionic and cationic space charge regions were found for each Hf–Nb–Ta alloy studied. Increasing the oxide thickness resulted in a current density plateau observable for all CV series at approximately 5 V (SHE). This plateau directly describes anodic oxide formation, and the amount of oxide grown per each volt applied (formation factor) can be calculated from its value as a material constant. Additionally, plotting the current densities measured during anodisation as a function of time (not shown here) allows the charge consumed by the oxidation process to be determined directly. The two approaches lead to identical results and allow mapping of the oxide formation factor across the investigated library via Faraday’s law.

**Figure 7.**
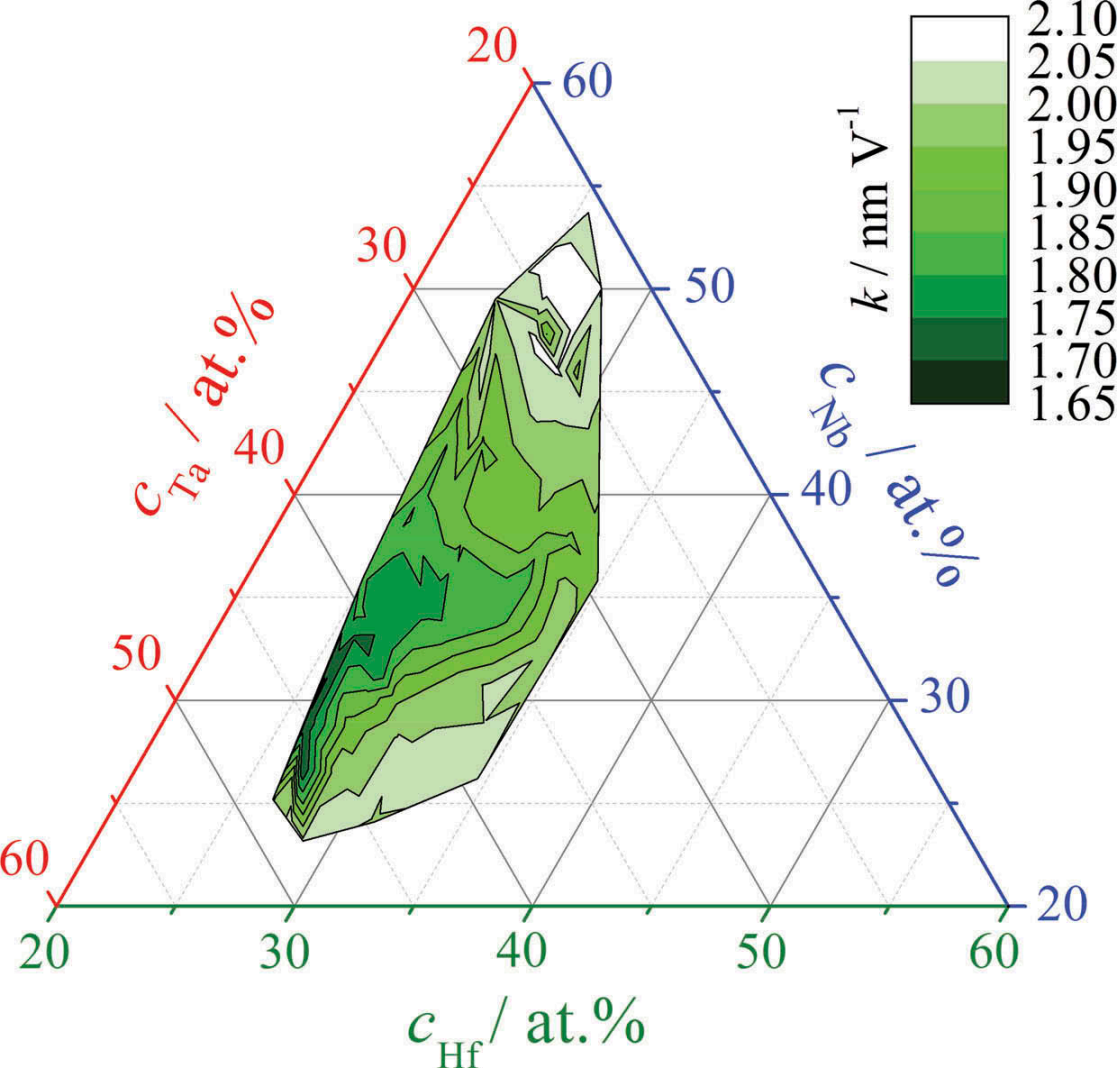
Oxide formation factor (in nm V^−1^) mapped as a material constant for the Hf–Nb–Ta thin film combinatorial library.

Assuming high current efficiency for the oxidation process, the oxide formation factors were calculated for each investigated alloy and the resulting mapping is presented in Figure 7. Using either coulometry or direct measurement of the current density plateaus (see Figure 6), the calculation included density values of individual Hf–Nb–Ta mixed oxides. These were obtained in accordance with the mixed matter model by a linear combination of pure oxide density values with respect to the species atomic fractions. The values of pure oxide densities used were 9.68, 4.36 and 8.10 g cm^−3^ for HfO_2_, Nb_2_O_5_ and Ta_2_O_5_, respectively [11].

The compositional variation of the oxide formation factor across the library shows well defined regions with values ranging from 1.65 to 2.10 nm V^−1^ that are in the range of previously reported values [39]. The lowest value was identified for alloys that had both the lowest Hf and lowest Nb amounts in the ternary library. The oxide formation factor within this compositional region, centred at approximately 27 at.% Hf, 30 at.% Nb and 43 at.% Ta, increases rapidly toward 2 nm V^−1^ with increasing Hf amount. However, above 30 at.% Nb the factor increases much more slowly with increasing Hf, slightly resembling the OCP mapping from Figure 5(b). The highest oxide formation factor was measured for alloys with the highest Nb and lowest Ta concentrations. These trends can be understood when the oxide formation factors reported for the anodisation of pure Hf, Nb and Ta in acetate buffer electrolyte (2.30, 2.60 and 1.80, respectively) are considered [23–25]. The highest amount of Ta in the library, where the smallest factor for the ternary alloys was identified, coincided with the lowest amounts of Hf and Nb. This is probably a result of the oxide formation factor of Ta being lower than those of Hf and Nb. Further, alloys with the highest formation factor were found at high concentrations of both Hf and Nb, possibly due to their high individual factors of more than 2 nm V^−1^. Interestingly, the lowest oxide formation factor measured for the ternary alloys had a value smaller than the lowest one of the individual metals, that is 1.80 nm V^−1^ for Ta. This suggests again that synergetic effects are present in the Hf–Nb–Ta library that is most likely related directly to the previously discussed synergy identified by the microstructure and crystallographic changes.

After each incremental anodisation step, each oxide grown on each Hf–Nb–Ta alloy addressed by SDCM was investigated using electrochemical impedance spectroscopy (EIS). The step-wise anodisation discussed previously (see Figure 6) allows EIS to be conducted as a function of anodic oxide thickness. Oxide properties can therefore be mapped across the entire library by properly fitting the EIS data. During all electrochemical measurements of anodised Hf–Nb–Ta alloys over a wide frequency range, a phase shift of close to −90° was found, while the impedance modulus always exhibited a slope of close to −1 when represented as a Bode plot (not shown here). These are strong indicators that a simple R_ox_C_ox_-R_el_ equivalent circuit can be used for data fitting, where the oxide resistance R_ox_ is taken in parallel to its capacitance C_ox_ and both together are taken in series with the electrolyte resistance R_el_. Such a fitting was performed for each ternary alloy analysed. As a result, at each location across the ternary library, oxide capacitance and resistance were obtained as functions of the anodisation potential. Further, using of the previously mapped oxide formation factor allowed a straightforward relation between the anodisation potential and oxide thickness to be defined. With this information, the simple definitions of capacitance and electrical resistance can be used to obtain the dielectric constant and electrical resistivity, respectively. All linear fitting procedures necessary for this action were performed automatically within the LabView software (developed in house) responsible for SDCM operation and data acquisition.

**Figure 8.**
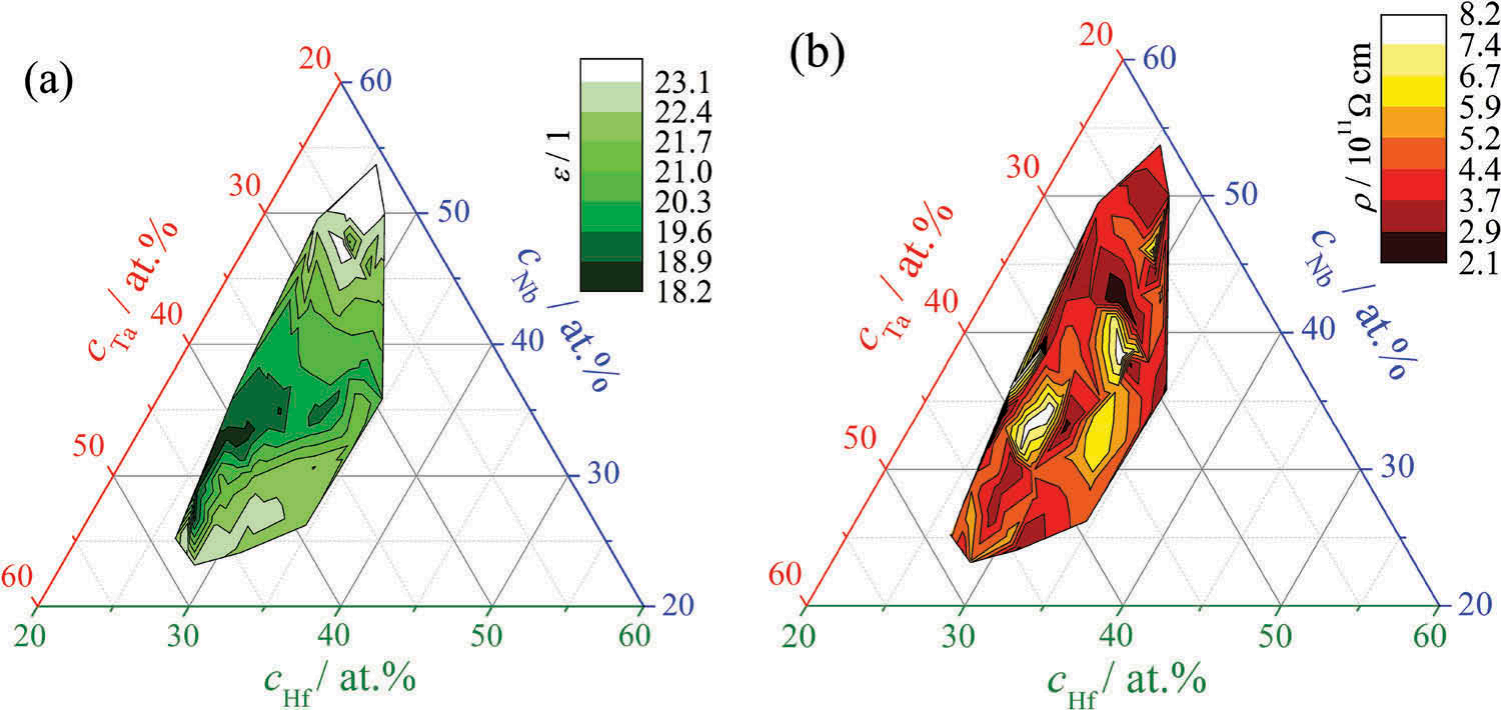
Compositional mapping of electrical permittivity (a) and electrical resistivity (b) of anodic oxides grown on Hf–Nb–Ta library.

The mappings of electrical permittivity and resistance of anodic oxides grown across the Hf–Nb–Ta library are presented in Figure 8. The colour-coded ternary diagram describing the compositional evolution of the dielectric constant presented in Figure 8(a) strongly resembles the compositional mapping of the oxide formation factor (see Figure 7). A minimum value of approximately 18 was measured in the compositional region with the lowest oxide formation factor. This relationship is defined mainly by the highest amount of Ta present in this region, since both Hf and Ta pure oxides have much lower permittivities than to Nb oxides (19 and 22 *vs*. 52) [23–25]. However, the permittivity mapping of the library is also directly affected by the measured oxide thickness values (the measured capacitance is inversely proportional to the thickness). This means that a resemblance to the thickness profiles across the entire library can be expected. If below 30 at.% Nb, a sharp increase in the dielectric constant with decreasing amount of Hf can be observed, above 30 at.% Nb this increase is strongly attenuated which results in a slight resemblance to the OCP mapping (see Figure 5(b)). The most important factor in the variation of permittivity is the Nb amount, most likely due to the permittivity of Nb_2_O_5_ being much higher than those of HfO_2_ and Ta_2_O_5_. However, the dielectric constant maximum measured in the library under study had a value of only slightly above 23 at around 50 at.% Nb. A strong influence of Ta is observed at low Nb concentrations around 25 at.% combined with Hf amounts between 28 and 33 at.%. Here the permittivity of mixed anodic oxides reaches values above 22 in spite of the low Nb content.

In part (b) of Figure 8 is presented the compositional mapping of the electrical resistivity of the anodic oxides as obtained from EIS data fitting. The values measured varied by almost one order of magnitude in the range of 10^11^–10^12^ Ω cm, which indicates the electrical behaviour of a dielectric (i.e. neither a very good insulator nor a very good semiconductor). Previous reports indicated resistivity values of 7.3, 2.3 and 4.8 · 10^11^ Ω cm for pure Hf, Nb and Ta oxides, respectively [23–25]. The values measured for the mixed Hf–Nb–Ta oxides across the library under study remain in the range of individual oxides resistivities (anodically grown on pure Hf, Nb and Ta).

The two extremes of this range defined by Nb and Hf may still correspond to a semiconductor or an insulator, respectively. Unlike various mappings already discussed, the resistivity mapping across the Hf–Nb–Ta library appears to be largely unaffected by the individual properties of pure oxides. The highest amount of Hf resulted in electrical resistivities below 5 × 10^11^ Ω cm in spite of the insulating behaviour of HfO_2_. Nevertheless, increasing the Nb content resulted in an overall increased conductivity. Two zones with insulating properties were identified centred approximately at 28 at.% Hf, 33 at.% Nb, 39 at.% Ta and at 30 at.% Hf, 39 at.% Nb, 31 at.% Ta, while the best electrical conductivity in the low 10^11^ Ω cm range was identified for 29 at.% Hf, 42 at.% Nb, 29 at.% Ta. The oxides in this region show better conductivity than Nb_2_O_5_ which again suggests a specific synergy of the Hf–Nb–Ta mixture during anodic oxide growth.

**Figure 9.**
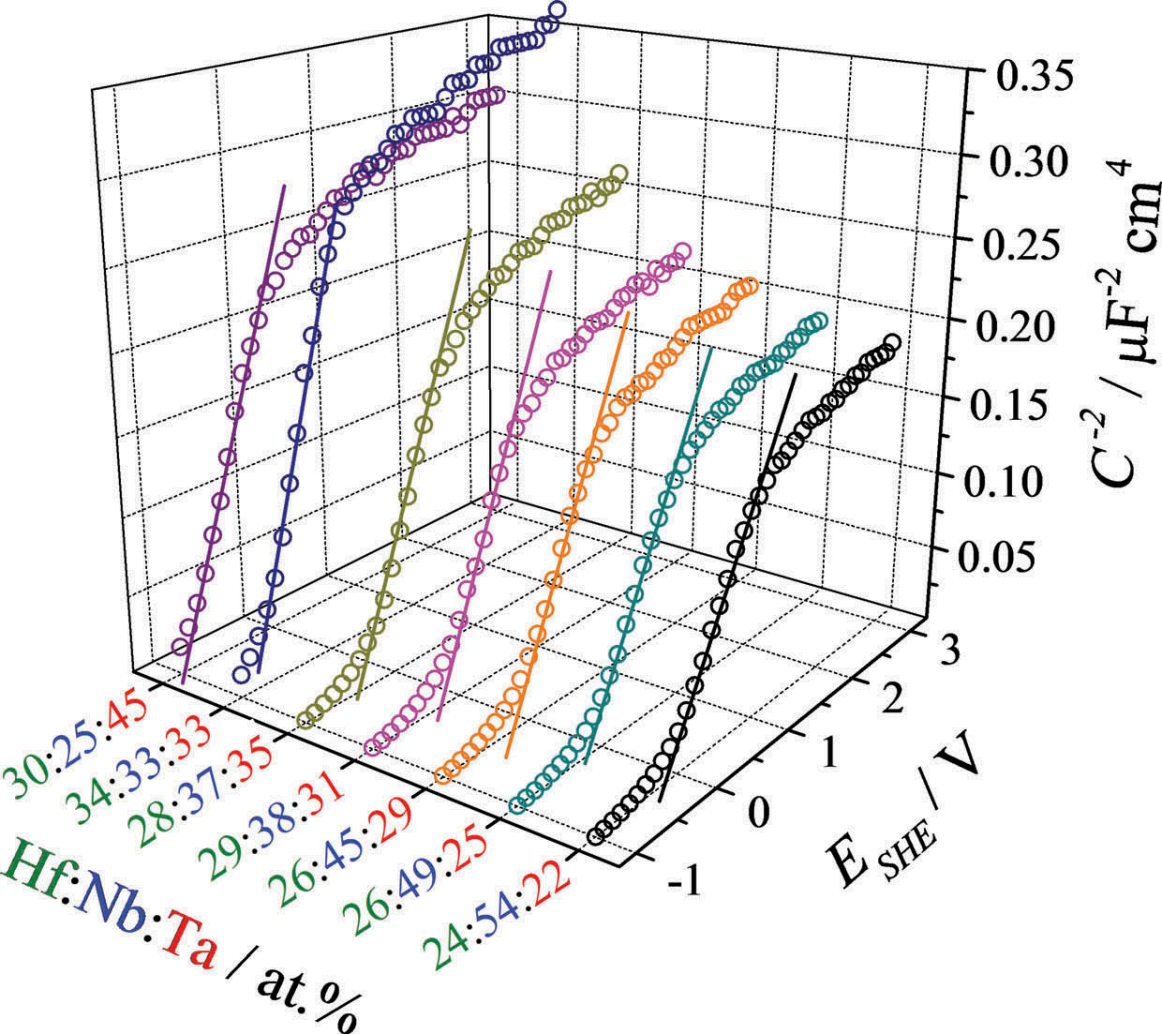
Selected Mott–Schottky plots for anodic oxides grown at 3 V (SHE) on various Hf–Nb–Ta thin film alloys.

To better characterise the semiconducting oxides across the Hf–Nb–Ta compositional spread, anodisations at 3 V (SHE) followed by Mott–Schottky analysis were performed using the SDCM. Remarkably, all oxides studied showed Mott–Schottky behaviour and several selected curves are presented in Figure 9. Scanning the applied bias from −1 V SHE to 3 V resulted in linear regions with negative slopes that are defined between inverse squared capacitance plateaus located at low and high biases. This is best observed at high Nb concentrations, most likely due to the semiconducting behaviour of pure Nb oxide. However, even at low Nb amounts a linear region can be identified. These linear regions indicate direct proportionality between inverse squared capacitance and bias. Their negative slopes describe negative majority charge carriers suggesting n-type semiconducting properties of the Hf–Nb–Ta anodic oxides.

**Figure 10.**
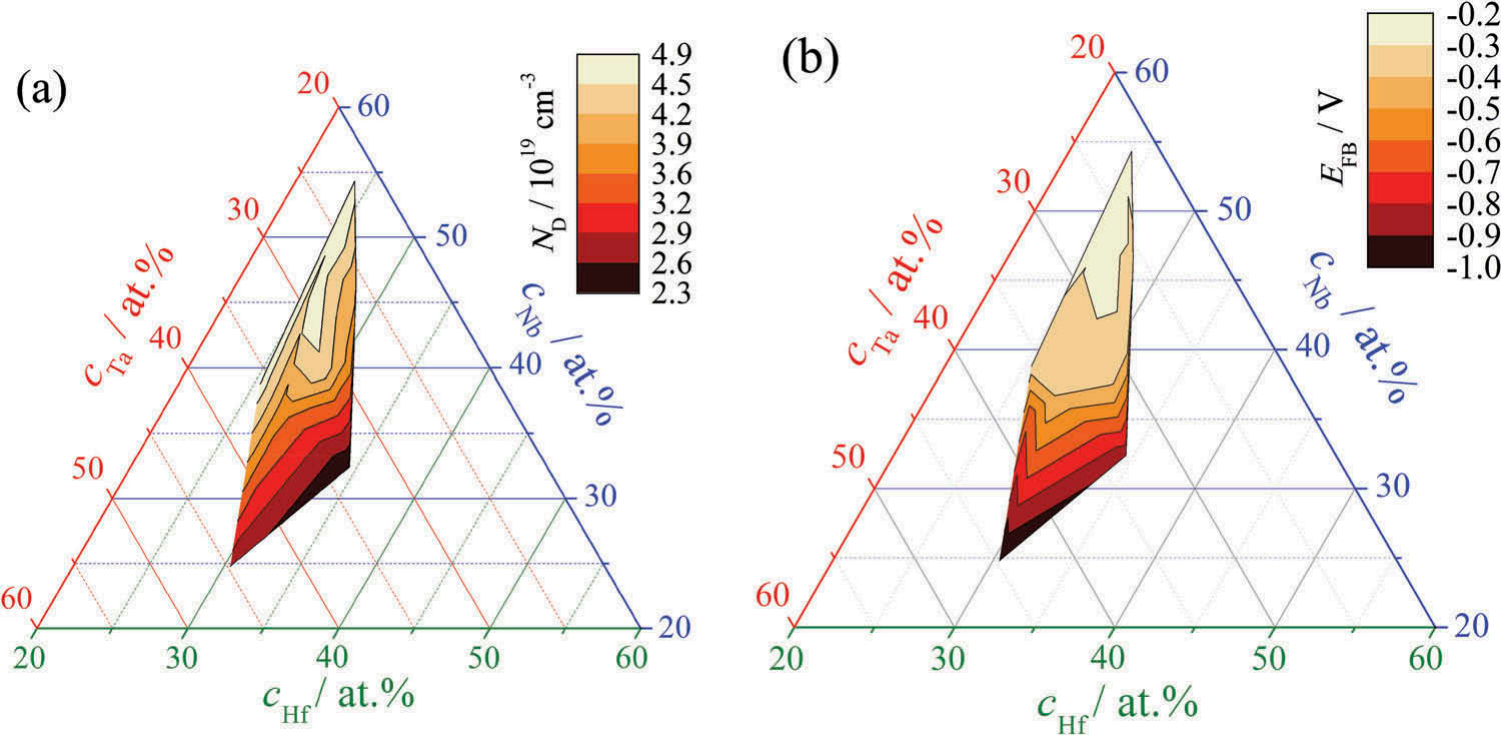
Carrier density (a) and flat-band potential (b) mappings for anodic alloys grown on Hf–Nb–Ta thin film library.

The slope and potential axis intercept values of Mott–Schottky plots allow the donor densities and flat-band potentials of each Hf–Nb–Ta oxide analysed to be calculated. Such a fitting procedure was performed for all Hf–Nb–Ta alloys studied and the resulting property mappings are presented in Figure 10. The donor density mapped in Figure 10(a) clearly indicates a behaviour triggered by the Nb amount in the mixed oxides. Around 50 at.% Nb, the highest donor densities of almost 5 × 10^19^ cm^−3^ can be found. This value decreases with decreasing Nb amount, and around 30 at.% Nb a value of about 10^18^ cm^−3^ is obtained. However, close examination of Figure 10(a) shows that Nb is not the only factor affecting the donor density of the mixed anodic oxides. Increasing the Hf amount also leads to decreasing carrier concentration, while varying the Ta content has almost no influence on the carrier concentration. This can be concluded directly from the colour mapping zones that are almost parallel to the Ta compositional axis. The highest dielectric constant and conductivity of all oxides studied were also found in the compositional zone with the highest carrier density (around 50 at.% Nb) in previous mappings. This is probably not a coincidence and again points to the behaviour of pure Nb oxide.

The amount of band bending due to alignment of the electrolyte redox level and the mixed anodic oxide Fermi level was evaluated for each composition. The flat-band potential mapping of the library is presented in Figure 10(b). The flat-band potential distribution across the library agrees well with the donor density compositional mapping. High Nb concentration in the parent metal alloys resulted in flat-band potentials slightly below 0 V. This may suggest a relatively straightforward charge transfer mechanism that can be exploited in future applications. Decreasing the Nb amount down to 37 at.% slightly affects the band bending while keeping the Fermi level of the ternary oxides close enough to the electrolyte redox level. Beyond this concentration, however, the absolute value of the flat-band potential rapidly increases with decreasing Nb amount and reaches a value of −1 V at the lowest Nb concentration. The shape of the colour mapping zones around 37 at.% Nb suggests that variation of the Hf amount has little influence on the band bending. This, however, changes when the Nb amount decreases further, most likely due to an increase in the Ta concentration from 35 to 45 at.%. This conclusion is consistent with the reported flat-band potential of Ta_2_O_5_ being as low as −0.8 V [40].

Due to the complex interpretation of the results collected from screening ternary compositional spreads, conclusions regarding the compositional evolution of physical/chemical properties are not always easily obtained. However, mapping of various properties across the Hf–Nb–Ta thin film combinatorial library allows identifying here certain compositions that show remarkable values. These values were directly taken from observation of all figures discussed in this study and are summarised in Table 1. These individual ternary alloys may represent a starting point for future individual studies, when maximising or minimising the value of a certain material property is requested. For example, the alloy with the highest nobility has almost the same composition as the alloy which in its oxidised form has the highest donor density. Such alloy may be used for semiconducting devices in harsh/corrosive environments. The metallic alloys producing oxides with highest and lowest dielectric constant (and oxide formation factor) have the same amount of Hf (26 at.%). This means that for applications such as high-k materials or capacitors, the amount of Nb and Ta play the major role. Similarly, for applications where the mixed oxide conductivity is a key factor the amount of Hf (28 at.%) plays a small role as compared to the Nb and Ta. Such applications may be thought in the direction of hot electron sources or tunnelling barriers. Screening of the oxide flat band potential may be useful in semiconductor applications where band matching, for example with a p-type semiconductor is crucial for carrier transport in a p–n junction. Here, applications toward solar energy conversion and water splitting may be further investigated.

**Table 1.** Relevant compositions resulting from screening of the Hf–Nb–Ta library corresponding to lowest and highest values of the mapped property.

	Hf:Nb:Ta/at.%	
Screened property	Lowest	Highest
Metal crystallinity	32:27:41	–
Metal electrical resistivity	25:38:37	34:35:31
Metal nobility	34:35:31	25:48:27
Oxide formation factor	26:30:44	26:50:34
Oxide dielectric constant	26:30:44	26:50:34
Oxide electrical resistivity	28:43:29	28:33:39
Oxide carrier density	34:32:34	25:50:25
Oxide flat band potential	31:26:43	25:50:25

## 4. Conclusions

A ternary thin film combinatorial library containing Hf, Nb and Ta was deposited by co-sputtering to characterise the fundamental properties in the Hf–Nb–Ta system. Microstructure and crystallographic mappings as functions of library composition revealed similarities in alloy evolution. Surface grains that are well grouped in domains with clearly defined boundaries were attributed mainly to mixing cubic Nb with tetragonal Ta. Addition of hexagonal Hf resulted in pronounced lattice distortion, yielding amorphous alloys above 32 at.% Hf and below 27 and 41 at.% Nb and Ta, respectively. This effect triggered morphological changes, specifically the disappearance of surface domains concomitant with surface smoothing. Volta and open circuit potential mappings of the surface indicated minimal values for the highest Hf concentration. Resistivity measurements mapping of the alloys revealed a strong dependency of Hf concentration, simultaneous with a minor contribution from both Nb and Ta. Library anodisation by scanning droplet cell microscopy revealed valve metal behaviour for all alloys and allowed electrochemical characterisation of the Hf–Nb–Ta mixed oxides. Oxide formation factors above 2 nm V^−1^ were found at high Nb and Ta concentrations. Electrochemical impedance spectroscopy allowed electrical permittivity and resistivity of mixed oxides to be mapped. Their compositional variations were linked to properties of the parent metal alloys and to particularities of individual pure oxides. A Mott–Schottky analysis revealed n-type semiconductor properties for all Hf–Nb–Ta oxides studied. Carrier concentration and flat-band potential were compositionally mapped and their values found to be depending mainly on the Nb amount. Synergetic effects were observed in various mappings of Hf–Nb–Ta parent metals and their anodic oxides throughout the library.
