# Supplementary figures and images for: Basic properties mapping of anodic oxides in the hafnium–niobium–tantalum ternary system
Source: Sci Technol Adv Mater. 2018 Aug 13;19(1):554–68. doi: 10.1080/14686996.2018.1498703 (PMC6095020; doi:10.1080/14686996.2018.1498703)

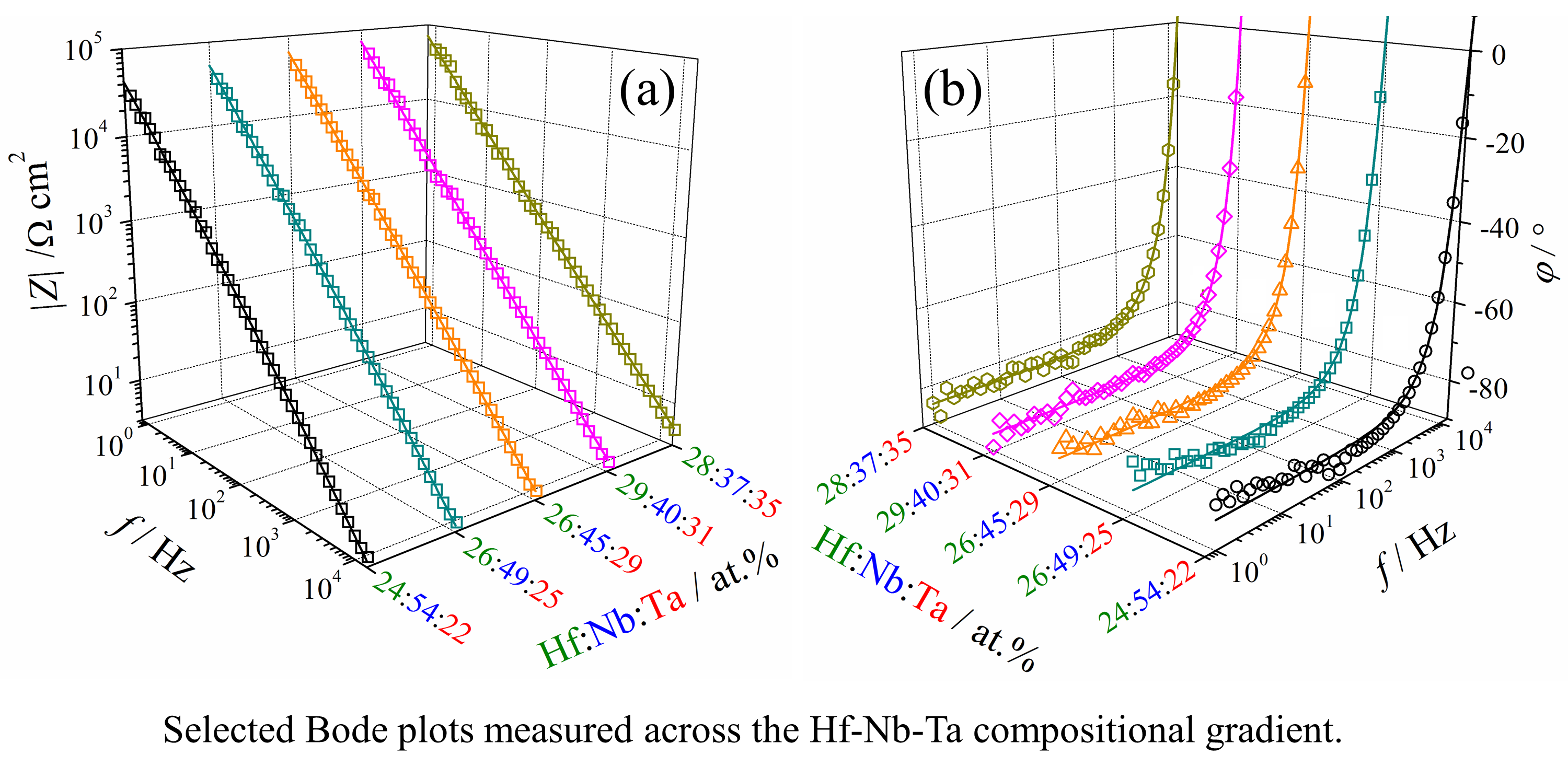

Supplement: Supplemental Material [file TSTA_A_1498703_SM7425.tif]
